# Impact of Titanium Skull Plate on Transcranial Magnetic Stimulation: Analysis of Induced Electric Fields

**DOI:** 10.3390/life14050642

**Published:** 2024-05-17

**Authors:** Mai Lu, Shoogo Ueno

**Affiliations:** 1 Key Laboratory of Opto-Electronic Technology and Intelligent Control of Ministry of Education, Lanzhou Jiaotong University, Lanzhou 730070, China; 2 Department of Biomedical Engineering, Graduate School of Medicine, The University of Tokyo, Tokyo 113-0033, Japan; uenos6325@gmail.com

**Keywords:** transcranial magnetic stimulation, titanium skull plate, realistic head model, figure-of-eight coil, impedance method

## Abstract

Background: Implanted titanium skull plates (TSPs) in cranioplasty are used to replace or reconstruct areas of the skull that have been damaged or removed due to trauma, surgery, or other medical conditions. However, the presence of a TSP in the head may influence the distribution of the electric field induced during transcranial magnetic stimulation (TMS) procedures. The purpose of this study was to determine how the presence of TSP would interfere with TMS-induced cortical electric fields. Methods: The TMS with a figure-of-eight coil was applied to a realistic head model with TSPs. The distribution of the induced electric field in head tissues was calculated by employing the impedance method, and the results were compared with that of a normal head without TSP. Results: Simulation results show that the distribution of the induced electric field has changed greatly for the head model with TSP. The maximum value of the induced electric field in head tissues was present under one of the circular coil wings rather than in the tissues beneath the junction of the two wings of the Fo8 coil. Conclusions: The induced electric field in deep brain regions was increased for the head model with TSP, which could potentially lead to deep brain stimulation. Since the presence of metallic TSP can greatly influence the distribution of the induced electric field in TMS applications, it is important to adjust the treatment scheme when considering TMS for individuals with cranial titanium plates.

## 1. Introduction

Transcranial magnetic stimulation (TMS) is a noninvasive technique used to stimulate the brain. It involves the use of strong magnetic fields generated by a coil placed on the scalp, which can induce electrical currents in the brain tissue, leading to the modulation of neuronal activity [1,2]. TMS has been used in various research and clinical applications to study brain function and investigate neurological and psychiatric disorders [3]. In research, TMS is used to study brain function and connectivity, investigate the mechanisms underlying neurological and psychiatric disorders, and develop new treatments [4]. In clinical practice, TMS has been approved by regulatory agencies for the treatment of major depressive disorder and is being investigated as a potential treatment for other conditions such as schizophrenia [5], obsessive compulsive disorder [6], and chronic pain [7,8,9]. TMS is considered a safe and well-tolerated procedure when performed according to established guidelines and safety protocols [10].

Due to traumatic injuries, congenital deformities, decompressive craniectomies, or bone flap loss resulting from infections, cranioplasty, a neurosurgical procedure, is performed to repair skull defects, aiming to achieve both cosmetic and functional improvements [11]. TSPs are commonly used in cranioplasty procedures due to their biocompatibility and strength. The procedure involves the reconstruction of a missing portion of the skull using titanium plates [12,13]. Cranioplasty is important for restoring the protective and aesthetic functions of the skull and is often performed to the improve neurological outcomes and quality of life of patients.

However, patients who have undergone cranioplasty may face mental health challenges. Depending on the surgical site and any complications, patients may encounter cognitive changes, memory issues, or concentration difficulties. The stress of a major surgical procedure, concerns about recovery, and potential complications can all contribute to anxiety, depression, and other mood disorders [14].

When considering the safety of metal object in the presence of electromagnetic fields, such as those generated during TMS, the generation of eddy currents and potential heating effects are important factors to consider [15]. Research has shown that the conventional low-frequency TMS protocols are unlikely to cause appreciable heating of the TSP or aneurysm clips surrounding brain tissues [16,17,18,19]. However, with the introduction of titanium alloy in the skull, the conductivity and geometry of the implant can affect the flow of induced currents, leading to changes in electric field distribution within the brain tissues [20]. Additionally, the presence of TSP may result in localized changes in the electric field strength, potentially impacting the targeted neural tissue and the overall effectiveness of the TMS treatment. This is an important issue, but it has rarely been considered in the literature [21,22]. In the present study, the impedance method was employed to numerically calculate the electric field induced in a realistic head model with TSP using a figure-of-eight (Fo8) coil. The characteristics of an induced electric field (E-field) in a head model and in deep brain tissues were investigated, and the results were compared with that of a normal head model without the titanium alloy.

## 2. Materials and Methods

**Figure 1:**
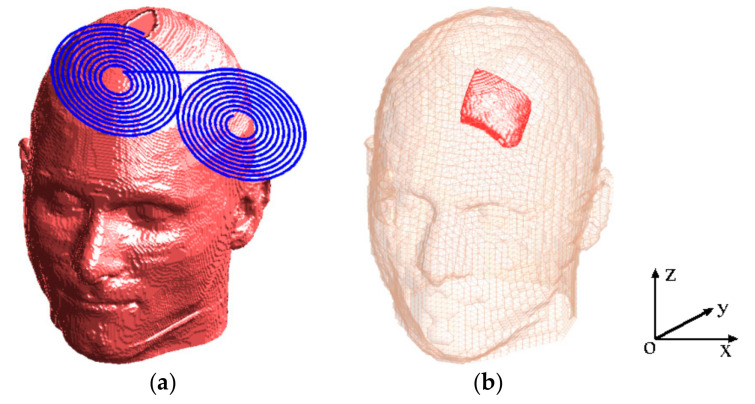
The head model with a Fo8 coil (**a**) and the head model with a titanium skull plate (**b**).

The realistic head model with a Fo8 TMS coil is shown in Figure 1a, and the head model with TSP is shown in Figure 1b. The head model was generated from a male model (Duke, 34-year-old male) developed under the Virtual Family project (Zurich, Switzerland), which consists of four anatomical resolution models based on magnetic resonance imaging (MRI) data of two adults and two children [23]. The male model has been segmented into 77 tissues, out of which 36 tissues were included in the present head model. The size of the head model is 188 mm × 240 mm × 232 mm in x-, y-, and z-directions, respectively. It is composed of more than 10 million cubic voxels with resolutions of 1 mm. The head model encompasses crucial brain subregions, including the hippocampus, midbrain, pons, pineal body, thalamus, etc. A titanium plate with a conductivity of 5.0 × 10^5^ S/m was used in the present study. We evaluated two sizes of TSP—4 cm × 4 cm and 6 cm × 6 cm, respectively—that are used for skull injuries.

The Fo8 coil was composed of two circular coil wings. Each circular wing had an inner radius of 10 mm and an outer radius of 50 mm. There are 10 wire turns in each wing. The coil was supplied with identical pulse currents at an amplitude of I = 5.0 kA and an operating frequency of 2.381 kHz.

The electrical conductivity of head tissues is determined using the four Cole–Cole model parameters [24]. This model characterizes how head tissues respond to an electric field with angular frequency through relaxation theory, enabling the calculation of tissue conductivity by fitting the model to experimental data [25,26,27]. In the current head model, which includes more tissue types than the original Gabriel list, different tissues in the head model are represented with conductivities matching those of similar tissues. For instance, tissues such as the thalamus, hippocampus, and pons are assigned the conductivity values of brain grey matter. The conductivities of head tissues and the titanium skull plate at a frequency of 2381 Hz used in the simulations are presented in Table 1.

When the head is subjected to a time-varying magnetic field, it can generate electric currents within the brain tissue according to Faraday’s law of electromagnetic induction. The impedance method can be employed to calculate these currents [28]. In this method, the head model is represented using a regular 3D Cartesian grid and consists of small cube-shaped voxels with dimensions specified as (nx, ny, and nz). Each voxel has dimensions of 1 mm × 1 mm × 1 mm. Within each voxel, the electric conductivities are isotropic and constant in all orientations. The model is depicted as a three-dimensional grid of impedances. The magnetic fields are computed employing Biot–Savart’s law, the induced currents are determined using the impedance method, and the induced electric fields are obtained utilizing Ohm’s law. The impedance method has proven to be a highly effective numerical technique for computing induced current densities and/or electric fields in anatomically voxel models when exposed to low-frequency electromagnetic fields [29,30].

## 3. Results

**Figure 2:**
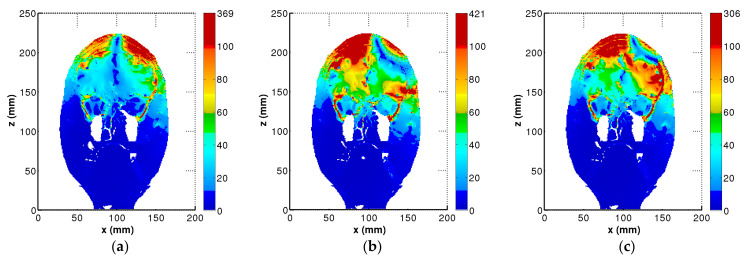
Electric field distributions in a coronal slice of y = 80 mm. (**a**) Head model without TSP, (**b**) head model with TSP-4 cm, and (**c**) head model with TSP-6cm.

Figure 2a–c shows the distribution of the E-field in a coronal slice of y = 80 mm for the normal head, the head model with a TSP of 4 cm × 4 cm (TSP-4 cm), and the head model with a TSP of 6 cm × 6 cm (TSP-6 cm), respectively. In order to show the results more clearly, nonlinear color bars are employed in these figures. The color scale covers the range 0–100 V/m and all values above 100 V/m (threshold for neuronal excitation [31]) are shown in dark red.

For the normal head model without implanted TSP, the E-field was mainly distributed under the head tissues beneath the coil center (Figure 2a). Comparatively, the distribution of the E-field changed greatly for the head model with either TSP-4cm or TSP-6cm. The maximum value of the E-field occurred in head tissues under the left circular wing, rather than at the junction of the two wings (Figure 2b,c). In addition, the E-field in deep brain regions also increased for the head model with TSP. It can be observed that the larger size of the TSP, the larger the E-field in deep brain regions.

**Figure 3:**
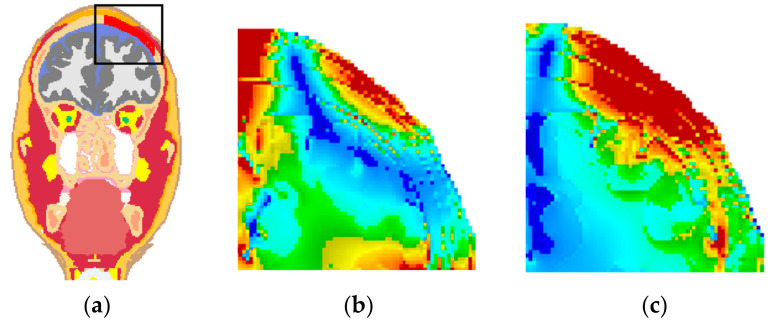
Comparison of E-field distribution in head tissues near the TSP. (**a**) Partially enlarged drawing of tissues, (**b)** head model with TSP-4cm, and (**c**) head model without TSP.

In order to highlight the detailed E-field distribution in head tissues near the TSP, partially enlarged drawings of the tissues in the small black box in Figure 3a are provided: the detailed E-field distribution in head tissues near the TSP is presented in Figure 3b; for comparison purpose, the E-field distribution in head tissues without the TSP is presented in Figure 3c. It can be clearly seen that the presence of the TSP can greatly alter the distribution of the induced electric field in the head. The E-field in the targeted brain regions, i.e., under the coil center, decreased greatly for the head model with TSP-4cm in Figure 3b compared with that in Figure 3c for the normal head model without TSP. The localized changes in the electric field strength potentially impact the targeted neural tissue and the overall effectiveness of the TMS treatment.

**Figure 4:**
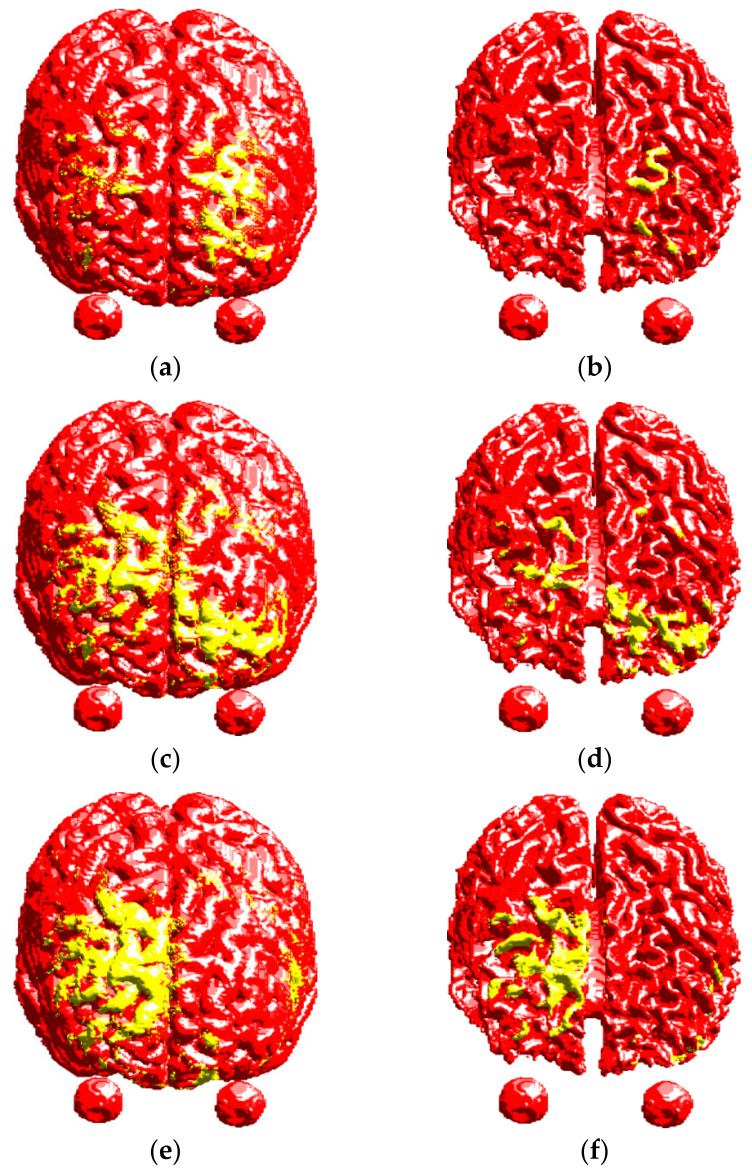
Electric field distributions on the cortical surfaces. Top row: head model without TSP, (**a**) GM and (**b**) WM. Middle row: head model with TSP-4cm, (**c**) GM and (**d**) WM. Bottom row: head model with TSP-6cm, (**e**) GM and (**f**) WM.

Figure 4 shows the 3D distribution of the E-field on the surfaces of gray matter (GM) and white matter (WM). Magnitudes of the E-field larger than 100 V/m are represented by yellow color, and the GM and WM surfaces are represented by red color. For the head model without TSP, the E-field was mainly distributed on the GM and WM surfaces of the right hemisphere with good focality (Figure 4a,b). For the head model with TSP-4cm, the E-field was mainly distributed on the GM and WM surfaces of the left hemisphere, and the focality became worse (Figure 4c,d). With the increase in TSP size, the E-field in the cortex under the center of the Fo8 coil decreased, while the E-field on the GM and WM surfaces of the left hemisphere were remarkably increased (Figure 4e,f).

**Figure 5:**
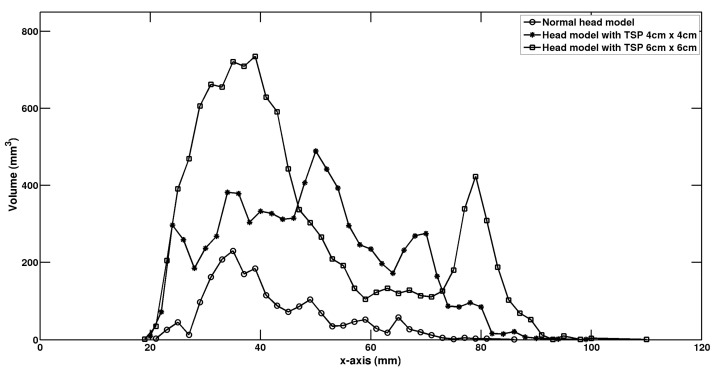
Relationship between the brain tissue volume with E > 100 V/m and the field penetration depths.

Figure 5 shows the dependence of brain volume with electric fields beyond 100 V/m on the distance from the vertex of the head. It can be found that the focal stimulation was present in superficial cortical regions for the normal head model without TSP. The depth of penetration also significantly improved, and a much slower rate of decay of the E-field as a function of the distance from the cortical surface for the head model with TSP was observed, which could potentially lead to deep brain stimulation for the head model with the TSP.

Table 2 displays the peak induced electric field in deep brain subregions for the head model with or without the TSP. It was found that the electric field in brain subregions was increased for the head model with the TSP. For example, the maximum induced electric field in the hippocampus is 30.3 V/m for the head model with TSP-4cm and 45.9 V/m for the head model with TSP-6cm, respectively—1.8 and 2.73 times larger compared with the that of normal head model, respectively.

**Figure 6:**
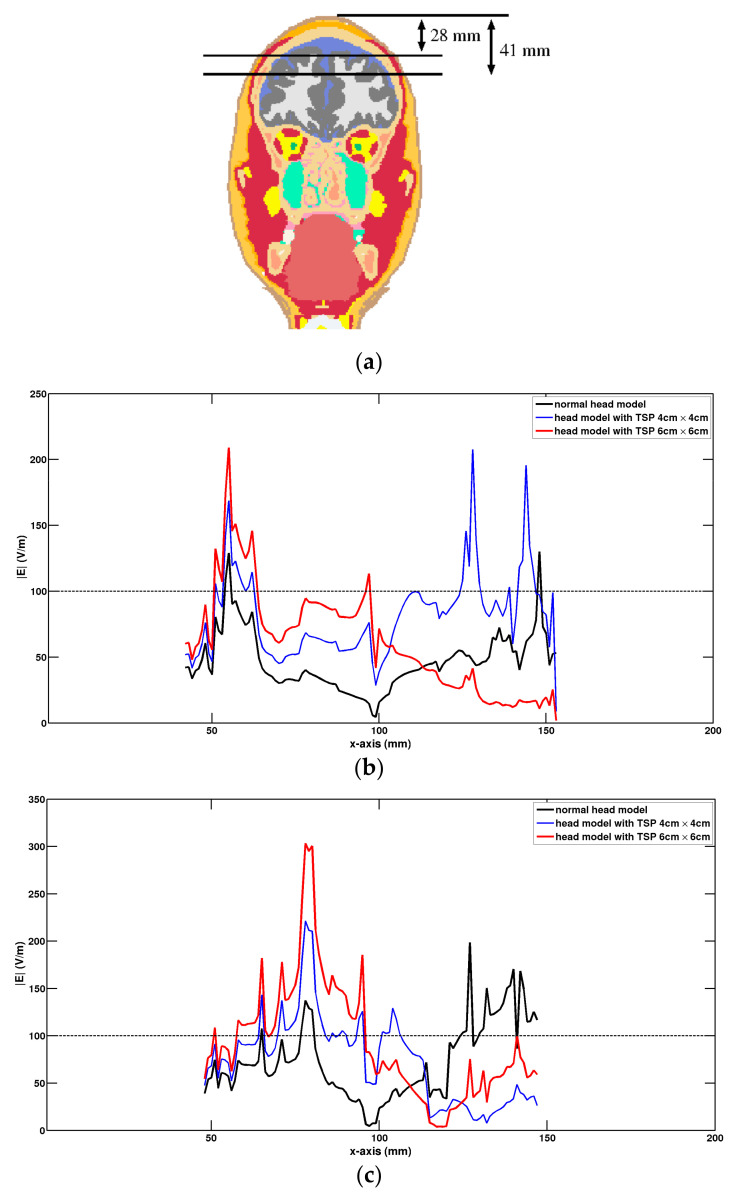
Comparison of electric field along the test lines at different depths. (**a**) Tissue slice at y = 80 mm (coronal plane) with two test lines, (**b**) test line is situated at a depth of 28 mm, and (**c**) test line is situated at a depth of 41 mm.

Figure 6 presents a quantitative comparison of the strength of the electric field along the test lines at different depths within brain tissues (Figure 6a) for the normal head and the head model with the TSP. For the case with a depth of 28 mm (Figure 6b), larger values of E-field were present in cortical regions under the left wing of the Fo8 coil for the head model with the TSP. For the normal head, the larger values of E-field were obtained under the junction area of the Fo8 coil. As the depth increased to 41 mm (Figure 6c), the electric field within deep brain regions was still above the threshold of 100 V/m in a wide area for the head model with the TSP.

## 4. Discussion

The literature on the behavior of titanium plates during transcranial magnetic stimulation (TMS) procedures provides valuable insights into heating and force considerations [16]. These references suggest that titanium plates exhibit minimal heating and low forces during TMS procedures [17]. However, with the introduction of titanium alloy into the skull, the conductivity and geometry of the implant can affect the flow of induced currents, leading to changes in electric field distribution within brain tissues [20]. In this paper, a realistic head model with a rectangular titanium skull plate was created. Three-dimensional distributions of the induced E-field in the head model with a TSP using Fo8 coil were obtained using the impedance method. The results were compared with that of the normal head model without a TSP. It was found that the induced electric field in the head model with the TSP changed greatly. The maximum value of the induced electric field was present under one of the circular coil wings rather than in the head tissues beneath the junction of the two wings in the Fo8 coil, resulting in poor stimulation focality. However, the induced electric field in deep brain regions was increased by introducing a TSP into the head model, which could potentially lead to deep brain stimulation.

Due to the potential impact of TSP on the propagation path and intensity distribution of the electric field, it is necessary to carefully consider the parameter settings and positioning of magnetic stimulation during the treatment process in practical clinical applications. These changes may affect the target area and efficacy of the treatment. Therefore, when conducting TMS therapy for patients with TSP implants, special attention should be paid to individual differences to ensure the effectiveness and safety of the treatment.

## 5. Conclusions

The efficacy of transcranial magnetic stimulation (TMS) in the presence of metallic titanium plates is an important issue. The presence of metallic TSP can influence the distribution of the induced electric field during TMS procedures, potentially affecting the depth of penetration and the focal nature of the stimulation via Fo8 coils. The results in this paper indicate that the normal TMS treatment scheme will not be suitable for patients with implanted TSPs in their heads. It is crucial to adjust the treatment scheme to account for the presence of TSPs in a patient’s head during TMS applications. This adjustment should be made on a case-by-case basis, taking into consideration the specific characteristics of the TSP, its location, geometry, size, etc. Numerical simulations can be used to predict and optimize the electric field distribution in the presence of a TSP, ensuring that the targeted brain regions receive the intended stimulation.

The limitation of this study lies in the fact that the human head model with an embedded titanium plate was too idealized, as we only used a healthy adult male head model and did not consider cortical lesions or injuries. We used a regular-shaped titanium plate, and did not consider irregularly shaped TSPs, or other shapes of TSP, such as round shapes and so on. These factors will affect the distribution of the induced electric field in brain tissue during TMS applications. To correctly account for all of these factors, a combination of a head model with a realistic skull injury and realistic titanium skull plate must be used.

## Figures and Tables

**Table 1:** Tissue conductivities.

Tissue	Conductivity (S/m)	Tissue	Conductivity (S/m)
Artery	7.00 × 10^−1^	Hypothalamus	5.26 × 10^−1^
Blood Vessel	3.10 × 10^−1^	Mandible	2.03 × 10^−2^
Cartilage	1.75 × 10^−1^	Marrow—bone	2.44 × 10^−3^
Cerebellum	1.24 × 10^−1^	MO*3	4.65 × 10^−1^
CSF	2.00 × 10^0^	Midbrain	4.65 × 10^−1^
CA*1	6.44 × 10^−2^	Mucosa	8.46 × 10^−4^
CP*2	6.44 × 10^−2^	Muscle	3.31 × 10^−1^
Connective Tissue	2.04 × 10^−1^	Nerve	3.04 × 10^−2^
Ear—cartilage	1.75 × 10^−1^	Pineal—body	5.26 × 10^−1^
Ear—skin	2.00 × 10^−4^	Pons	4.65 × 10^−1^
Eye—cornea	4.25 × 10^−1^	Skin	2.00 × 10^−4^
Eye—lens	3.31 × 10^−1^	Skull	2.03 × 10^−2^
Eye—sclera	5.07 × 10^−1^	Spinal Cord	3.04 × 10^−2^
Eye—vitreous humor	1.50 × 10^0^	Teeth	2.03 × 10^−2^
FAT	2.32 × 10^−2^	Thalamus	1.04 × 10^−1^
Gray matter	1.04 × 10^−1^	Tongue	2.76 × 10^−1^
Hippocampus	1.04 × 10^−1^	titanium	5.00 × 10^5^
Hypophysis	5.26 × 10^−1^	White Matter	6.44 × 10^−2^

CA*1: commissura—anterior; CP*2: commissura—posterior; MO*3: medulla oblongata.

**Table 2:** Maximum electric field (V/m) in brain subregions.

Tissue	Head Model with TSP-4cm	Head Model with TSP-6cm	Normal Head Model
Cerebellum	22.5	25	20.4
Commissure—Anterior	18.3	21.1	14.1
Commissure—Posterior	10.3	16.7	2.94
Hippocampus	30.3	45.9	16.8
Hypophysis	54.3	67.6	33.9
Hypothalamus	30.9	39.5	19.13
Medulla Oblongata	3.7	4.9	2.52
Midbrain	14.9	20.6	8.89
Pineal—Body	5.3	7.8	3.55
Pons	8.8	10.9	6.46
Thalamus	29.7	41.2	16.38

## Data Availability

All data are available upon request.
